# Menstrual cycles and macrocycles: Science, not socials, is doing the heavy lifting

**DOI:** 10.1113/JP288096

**Published:** 2025-02-25

**Authors:** Clare Minahan

**Affiliations:** ^1^ Griffith Sports Science Griffith University, Gold Coast QLD Australia; ^2^ Female Performance & Health Initiative Australian Institute of Sport Canberra ACT Australia

**Keywords:** female athletes, hypertrophy, phase‐based training, resistance training, sex steroids, women

## Introduction

The role of sex hormones in skeletal muscle adaptation is a subject of ongoing debate. Public perception often associates higher levels of testosterone with increased muscle size, centring the discussion around men. Less consideration has been given to female sex hormones, oestrogen and progesterone, and their influence on skeletal muscle adaptation. Fluctuations in oestrogen and progesterone across the menstrual cycle (MC) is a hallmark of female reproductive physiology, with increasing attention in the scientific and social media communities. It is often assumed that these sex hormones create distinct anabolic and catabolic environments across the MC, potentially affecting responses to resistance exercise. Such assumptions have arisen from anecdotal reporting, popular opinion, physiological theory, and some scientific evidence. In their article published in *The Journal of Physiology*, Colenso‐Semple et al. (2024) dismantle this narrative with precision and elegance. Their findings clearly demonstrate that fluctuations in sex hormones across the MC do not compromise the capacity of women to respond effectively to an appropriate resistance exercise stimulus. The results of the study by Colenso‐Semple et al. (2024) prompt a critical question: does the evidence justify ‘MC‐phase‐based’ resistance exercise training?

## Context and significance

Theories about MC phase and resistance exercise training outcomes often emphasize the late follicular phase, when oestradiol levels peak, as an anabolic phase favourable for muscle adaptation, compared to the mid‐luteal phase, where both oestradiol and progesterone are elevated (Oosthuyse et al., 2023). Animal studies have reported impaired muscle repair in the absence of oestrogen (Toth et al., 2001) but translating such findings to humans has been difficult as ovariectomy in animal models most probably does not accurately represent the cyclical hormonal changes of the human MC. Methodological inconsistencies, including small sample sizes, imprecise MC phase identification and reliance on acute protein synthesis measurements, have hampered human research. Evidence reviewed by Kissow et al. (2002) reflects mixed results. Some studies suggest potential benefits of resistance exercise training in the follicular phase, although their findings are limited by methodological constraints such as basal body temperature for phase verification. Other research reported improved lean muscle mass gains after follicular‐phase‐based training, but the inclusion of participants using oral contraceptives complicates interpretation of the results. Studies reporting no significant differences in resistance exercise training outcomes between MC phases support the notion that fluctuations in sex hormones do not influence skeletal muscle adaptation. Taken together, these findings challenge the assumption that MC phase significantly affects resistance exercise training outcomes. Colenso‐Semple et al. (2024) offer compelling evidence that muscle protein turnover is unaltered by MC phase. Their study reflects the methodological gold standard (Elliott‐Sale et al., 2021) and sets a new benchmark for future research: a within‐subject cross‐over trial with thorough phase verification using ovulation kits, hormonal assays and metabolomics. Their study finds no evidence to support the claim that resistance exercise training outcomes, as reflected by muscle protein synthesis and myofibrillar proteolysis, differ between the follicular and luteal phases of the MC.

## Implications for research and practice

Concerns about fluctuations in female sex hormones as a confounding factor have contributed to the underrepresentation of women in exercise science research. Evidence that MC phase does not significantly impact exercise responses should encourage researchers to move beyond this assumption, enabling more inclusive and cost‐effective study designs. Removing the need to tightly control for MC phases would also reduce participant exclusions and improve research timelines. For athletes and coaches, the practical implications are profound. The evidence challenges the necessity of tailoring resistance exercise training programs to specific MC phases, simplifying program design and empowering women to train effectively throughout their MC. Concerns that elevated progesterone during the luteal phase might impair skeletal muscle adaptation are unfounded, as shown by consistent myofibrillar protein turnover across MC phases. Although there may be no specific advantage to training during the follicular phase, neither is there evidence of detriment during the luteal phase. This insight simplifies training recommendations and promotes resistance exercise programming for women.

## Considerations and future directions

Although evidence suggests that the MC phase does not significantly affect muscle protein turnover, other factors associated with the MC may influence skeletal muscle adaptation. For example, fatigue, mood disturbances and pain, which are all common in the luteal phase of the McNamara et al. (2022), could reduce total work performed during resistance exercise training, thereby impacting long‐term outcomes. Additionally, psychological attributes such as confidence, perseverance and risk‐taking, which may vary with hormonal fluctuations, could indirectly influence resistance exercise training adaptations. As such, autoregulatory training practices that align with how women feel during various phases of the MC, namely energized *vs*. fatigued, could be one way for women to plan their resistance exercise training. For example, elevated testosterone concentrations during the follicular phase may enhance such attributes, supporting higher training intensity and consistency. Longitudinal studies examining resistance exercise training outcomes (e.g. skeletal muscle hypertrophy and strength adaptations) across multiple MC cycles are critical for understanding potential subtle effects of sex hormone fluctuations. Furthermore, including diverse populations, such as post‐menopausal women and individuals with MC disturbances, is necessary to ensure that findings are generalizable across different demographic groups. The role of synthetic hormones, including contraceptives, and the nuanced influence of testosterone on female athletes warrant further investigation to refine exercise training protocols and deepen understanding of female‐specific physiology. These considerations provide a foundation for advancing research into MC‐related factors and their interactions with resistance exercise. Such efforts will enhance evidence‐based practices, enabling the development of inclusive, individualized training strategies that accommodate physiological and psychological variability.

## Additional information

### Competing interests

No competing interests declared.

### Author contributions

Sole author.

### Funding

None.

## Supporting information
