# Supplementary material for: Menstrual cycles and macrocycles: Science, not socials, is doing the heavy lifting
Source: J Physiol. 2025 Feb 25;603(5):1023–4. doi: 10.1113/JP288096 (PMC11870042; doi:10.1113/JP288096)
Supplement: Supplementary file 1 [file TJP-603-1023-s001.pdf]

The Journal of Physiology

Menstrual cycles and macrocycles: Science, not socials, is doing the heavy lifting

Clare Minahan
DOI: 10.1113/JP288096

Corresponding author(s): Clare Minahan (c.minahan@griffith.edu.au)

The referees have opted to remain anonymous.

Review Timeline:

Submission Date:	10-Dec-2024
Editorial Decision:	18-Dec-2024
Revision Received:	27-Jan-2025
Accepted:	29-Jan-2025

Senior Editor: Karyn Hamilton

Reviewing Editor: Robert Musci

Transaction Report:

(Note: With the exception of the correction of typographical or spelling errors that could be a source of ambiguity, letters and reports are not edited. Depending on transfer agreements, referee reports obtained elsewhere may or may not be included in this compilation. Referee reports are anonymous unless the Referee chooses to sign their reports.)

Dear Dr Minahan,

Re: JP-P-2024-288096 "Menstrual cycles and macrocycles: Science, not socials, is doing the heavy lifting" by Clare Minahan

Thank you for submitting your manuscript to The Journal of Physiology. It has been assessed by a Reviewing Editor and by 1 expert referees and we are pleased to tell you that it is acceptable for publication following satisfactory revision.

Please advise your co-authors of this decision as soon as possible.

The referee reports are copied at the end of this email.

Please address all the points raised and incorporate all requested revisions or explain in your Response to Referees why a change has not been made. We hope you will find the comments helpful and that you will be able to return your revised manuscript within 4weeks. If you require longer than this, please contact journal staff: jp@physoc.org.

Your revised manuscript should be submitted online using the link in your Author Tasks Link Not Available. This link is accessible via your account as Corresponding Author; it is not available to your co-authors. If this presents a problem, please contact journal staff (jp@physoc.org). Image files from the previous version are retained on the system. Please ensure you replace or remove any files that are being revised.

If you do not wish to submit a revised version of your manuscript, you must inform our journal staff (jp@physoc.org) or reply to this email to request withdrawal. Please note that a manuscript must be formally withdrawn from the peer review process at one journal before it may be submitted to another journal.

TRANSPARENT PEER REVIEW POLICY: To improve the transparency of its peer review process The Journal of Physiology publishes online, as supporting information, the peer review history of all articles accepted for publication. Readers will have access to decision letters, including Editors' comments and referee reports, for each version of the manuscript, as well as any author responses to peer review comments. Referees can decide whether or not they wish to be named on the peer review history document.

ABSTRACT FIGURES: Authors are expected to use The Journal's premium BioRender account to create/redraw their Abstract Figures. Information on how to access this account is here: https://physoc.onlinelibrary.wiley.com/journal/14697793/biorender-access.

This will enable Authors to create and download high-resolution figures. If authors have used the free BioRender service, they can use the instructions provided in the link above to download a high-resolution version suitable for publication.
The link provided should only be used for the purposes of this submission. Authors will be charged for figures created on this account if they are not related to this manuscript submission.

LANGUAGE EDITING AND SUPPORT FOR PUBLICATION: If you would like help with English language editing, or other article preparation support, Wiley Editing Services offers expert help, including English Language Editing, as well as translation, manuscript formatting, and figure formatting at www.wileyauthors.com/eeo/preparation. You can also find resources for Preparing Your Article for general guidance about writing and preparing your manuscript at www.wileyauthors.com/eeo/prepresources.

REVISION CHECKLIST:
Check that your Methods section conforms to journal policy: https://jp.msubmit.net/cgi-bin/main.plex?form_type=display_requirements#methods

Check that data presented conforms to the statistics policy: https://jp.msubmit.net/cgi-bin/main.plex?form_type=display_requirements#statistics

Upload a full Response to Referees file. To create your 'Response to Referees' copy all the reports, including any comments from the Senior and Reviewing Editors, into a Microsoft Word, or similar, file and respond to each point, using font or background colour to distinguish comments and responses and upload as the required file type.

Please upload two versions of your manuscript text: one with all relevant changes highlighted and one clean version with no changes tracked. The manuscript file should include all tables and figure legends, but each figure/graph should be uploaded as separate, high-resolution files. The journal is now integrated with Wiley's Image Checking service. For further details, see: https://www.wiley.com/en-us/network/publishing/research-publishing/trending-stories/upholding-image-integrity-wileys-image-screening-service

You may also upload:
- 'Potential Cover Art' for consideration as the issue's cover image
- Appropriate Supporting Information (Video, audio or data set: see https://jp.msubmit.net/cgi-bin/main.plex?

form_type=display_requirements#supp).

We look forward to receiving your revised submission.

If you have any queries, please reply to this email and we will be pleased to advise.

Yours sincerely,

Karyn Hamilton
Senior Editor
The Journal of Physiology

EDITOR COMMENTS

Reviewing Editor:

Thank you for writing this Perspective.

As indicated by the Reviewer, it is well-written and informative and accessible to a broad audience.

The Reviewer offered some minor feedback to consider.

Senior Editor:

Thank you so much for writing this Perspective on a recent outstanding original research paper published in The Journal of Physiology. At this point we would like to provisionally accept your manuscript, but wanted to give you the opportunity to consider the Referee's feedback which suggests a minor edit. Also, to provide the readers with a clear "roadmap," I wonder if you would mind citing the original research paper for which you are providing a Perspective? Staff can help with that if you are missing the necessary information for the citation. Thank you again and we appreciate you interest in The Journal of Physiology.

REFEREE COMMENTS

Referee #1:

The author's perspective is a well-written and timely contribution. I think the tongue-in-cheek title is a clever play on words and addresses the 'controversy' around the cycle-syncing phenomenon: social media versus science. I have only a few suggestions that the author may wish to consider.

Lines 35-37 The author states that the translation of animal findings to humans has been difficult. I think it's actually impossible with the model of ovariectomy to translate that condition to anything other than a woman who has had a radical hysterectomy. Ovariectomy is certainly not a model of the cyclical change in menstrual cycle hormones.

It may be worth mentioning that autoregulatory training practices that align with how women feel - fatigued versus energized - would be a way for women to plan their resistance training.

END OF COMMENTS

Thank you for the positive comments. Please find responses to the feedback provided, below.

Senior Editor:

1. I wonder if you would mind citing the original research paper for which you are providing a Perspective?

   Author response:
   I did indeed cite and provide the details of original research paper in the main body of the perspectives piece. I am very happy if the journal would like to add this to the reference list.

Referee #1:

1. Lines 35-37 The author states that the translation of animal findings to humans has been difficult. I think it's actually impossible with the model of ovariectomy to translate that condition to anything other than a woman who has had a radical hysterectomy. Ovariectomy is certainly not a model of the cyclical change in menstrual cycle hormones.

   Author response:
   You are indeed accurate. This sentence acknowledges this and now reads:

   Animal studies have reported impaired muscle repair in the absence of oestrogen (Toth et al., 2001) but translating such findings to humans has been difficult as ovariectomy in animal models is unlikely to accurately represent the cyclical hormonal changes of the human MC.

2. It may be worth mentioning that autoregulatory training practices that align with how women feel - fatigued versus energized - would be a way for women to plan their resistance training.

   Author response:
   Thank you for this suggestion. This sentence has now been added to acknowledge this point:

   As such, autoregulatory training practices that align with how women feel during various phases of the MC—energized versus fatigued—could be one way for women to plan their resistance exercise training.

END OF COMMENTS

Dear Associate Professor Minahan,

Re: JP-P-2025-288096R1 "Menstrual cycles and macrocycles: Science, not socials, is doing the heavy lifting" by Clare Minahan

We are pleased to tell you that your paper has been accepted for publication in The Journal of Physiology.

The last Word (or similar) version of the manuscript provided will be used by the Production Editor to prepare your proof. When this is ready you will receive an email containing a link to Wiley's Online Proofing System. The proof should be thoroughly checked and corrected as promptly as possible.

- The reference list must be in alphabetical order, rather than numbered, to comply with our Journal format.

Authors should note that it is too late at this point to offer corrections prior to proofreading. Major corrections at proof stage, such as changes to figures, will be referred to the Editors for approval before they can be incorporated. Only minor changes, such as to style and consistency, should be made at proof stage. Changes that need to be made after proof stage will usually require a formal correction notice.
All queries at proof stage should be sent to: TJP@wiley.com.

Following copyediting and typesetting the accepted version will be published online.

Yours sincerely,

Karyn Hamilton
Senior Editor
The Journal of Physiology

IMPORTANT POINTS TO NOTE FOLLOWING YOUR PAPER'S ACCEPTANCE:
If you would like to receive our 'Research Roundup', a monthly newsletter highlighting the cutting-edge research published in The Physiological Society's family of journals (The Journal of Physiology, Experimental Physiology, Physiological Reports, The Journal of Nutritional Physiology, and The Journal of Precision Medicine: Health and Disease), please click this link, fill in your name and email address and select 'Research Roundup':
https://www.physoc.org/journals-and-media/membernews

• TRANSPARENT PEER REVIEW POLICY: To improve the transparency of its peer review process, The Journal of Physiology publishes online as supporting information the peer review history of all articles accepted for publication. Readers will have access to decision letters, including Editors' comments and referee reports, for each version of the manuscript as well as any author responses to peer review comments. Referees can decide whether or not they wish to be named on the peer review history document.

• You can help your research get the attention it deserves! Check out Wiley's free Promotion Guide for best-practice recommendations for promoting your work at: www.wileyauthors.com/eeo/guide. You can learn more about Wiley Editing Services which offers professional video, design, and writing services to create shareable video abstracts, infographics, conference posters, lay summaries, and research news stories for your research at: www.wileyauthors.com/eeo/promotion.

• IMPORTANT NOTICE ABOUT OPEN ACCESS: To assist authors whose funding agencies mandate public access to published research findings sooner than 12 months after publication, The Journal of Physiology allows authors to pay an Open Access (OA) fee to have their papers made freely available immediately on publication.

The Corresponding Author will receive an email from Wiley with details on how to register or log-in to Wiley Authors Services where you will be able to place an order

• You can check if your funder or institution has a Wiley Open Access Account here: https://authorservices.wiley.com/author-resources/Journal-Authors/licensing-and-open-access/open-access/author-compliance-tool.html.

EDITOR COMMENTS

Thank you for these final revisions--much appreciated! We appreciate your efforts and are pleased to accept this for publication in The Journal of Physiology. Looking forward to seeing work from your group again in the future.
